# Different transmembrane domains determine the specificity and efficiency of the cleavage activity of the γ-secretase subunit presenilin

**DOI:** 10.1016/j.jbc.2023.104626

**Published:** 2023-03-20

**Authors:** Fabian C. Schmidt, Katja Fitz, Lukas P. Feilen, Masayasu Okochi, Harald Steiner, Dieter Langosch

**Affiliations:** 1 Biopolymer Chemistry, Technical University of Munich, Freising, Germany; 2 German Center for Neurodegenerative Diseases (DZNE), Munich, Germany; 3 Neuropsychiatry, Division of Internal Medicine, Department of Integrated Medicine, Osaka University Graduate School of Medicine, Suita, Japan; 4 Division of Metabolic Biochemistry, Faculty of Medicine, Biomedical Center (BMC), Ludwig-Maximilians-University, Munich, Germany

**Keywords:** Alzheimer’s disease, gamma-secretase, intramembrane proteolysis, amyloid precursor protein (APP), amyloid-beta (Aβ), presenilin, transmembrane domain

## Abstract

The γ-secretase complex catalyzes the intramembrane cleavage of C99, a carboxy-terminal fragment of the amyloid precursor protein. Two paralogs of its catalytic subunit presenilin (PS1 and PS2) are expressed which are autocatalytically cleaved into an N-terminal and a C-terminal fragment during maturation of γ-secretase. In this study, we compared the efficiency and specificity of C99 cleavage by PS1- and PS2-containing γ-secretases. Mass spectrometric analysis of cleavage products obtained in cell-free and cell-based assays revealed that the previously described lower amyloid-β (Aβ)38 generation by PS2 is accompanied by a reciprocal increase in Aβ37 production. We further found PS1 and PS2 to show different preferences in the choice of the initial cleavage site of C99. However, the differences in Aβ38 and Aβ37 generation appear to mainly result from altered subsequent stepwise cleavage of Aβ peptides. Apart from these differences in cleavage specificity, we confirmed a lower efficiency of initial C99 cleavage by PS2 using a detergent-solubilized γ-secretase system. By investigating chimeric PS1/2 molecules, we show that the membrane-embedded, nonconserved residues of the N-terminal fragment mainly account for the differential cleavage efficiency and specificity of both presenilins. At the level of individual transmembrane domains (TMDs), TMD3 was identified as a major modulator of initial cleavage site specificity. The efficiency of endoproteolysis strongly depends on nonconserved TMD6 residues at the interface to TMD2, *i.e.*, at a putative gate of substrate entry. Taken together, our results highlight the role of individual presenilin TMDs in the cleavage of C99 and the generation of Aβ peptides.

γ-Secretase is an intramembrane protease which is known to cleave around 150 different substrates, all of which are type I single-spanning integral membrane proteins (1). Cleavage of C99, a proteolytic fragment of amyloid precursor protein (APP), generates a series of amyloid-β (Aβ) peptides. Since some Aβ peptides are widely believed to cause Alzheimer’s disease (AD) (2), C99 cleavage by γ-secretase arguably represents the most intensely investigated case of intramembrane proteolysis. The production of Aβ peptides by C99 cleavage is mainly initiated at alternative ε48- and ε49-sites within its TMD (3, 4, 5, 6). This endoproteolytic initial cleavage liberates the C-terminal APP intracellular domain (AICD). Proteolysis continues toward the N-terminus and releases predominantly tripeptides and tetrapeptides by cleaving alternative ζ- and γ-sites (7, 8). Thus, two alternative product lines can be distinguished, depending on whether cleavage is initiated at the ε48- or at the ε49-site. Processive cleavage along these product lines and some cross-over between them (9, 10, 11) generates Aβ peptides of different length and toxicity (12, 13, 14).

The γ-secretase enzyme complex contains four different subunits, presenilin (PS), nicastrin (NCT), presenilin enhancer 2 (PEN-2), and anterior pharynx defective (APH-1) at a 1:1:1:1 stoichiometry. PS is autoproteolytically cleaved into an N- and a C-terminal fragment (NTF, CTF) (15, 16, 17, 18, 19). The existence of different paralogs of PS (PS1, PS2) and APH (APH-1a, APH-1b) can result in at least four different γ-secretase complexes (reviewed in: (14, 20, 21)). The diversity of PS is of particular interest as it represents the enzymatic component of the γ-secretase complex. While PS1-containing γ-secretase is primarily routed to the plasma membrane, PS2 sorts the complex mainly to the *trans*-Golgi network and late endosomes/lysosomes (22, 23). Both paralogs share a 66.3% sequence identity at the amino acid level. A number of studies have identified functional differences between the different γ-secretase complexes. With regard to both PS paralogs, PS2 cleavage at ε-sites has been reported to be less efficient than cleavage by PS1 (22). Further, PS2-containing γ-secretase has been found to produce less total Aβ (22, 24, 25), Aβ40 and Aβ42 (22, 26), a lower Aβ42/Aβ40 ratio (27), less secreted Aβ38 relative to Aβ40 (27, 28), and lower levels of Aβ38 and Aβ42 (29) compared to γ-secretase containing PS1. The picture emerging from those studies indicated that PS2-containing γ-secretase is less efficient in endoproteolysis and differs in exoproteolysis-like trimming relative to its counterpart harboring PS1.

It had remained unclear, however, how the identity of the PS paralog affected the specificity of ε-cleavage and which protein domains are responsible for the different cleavage activities of PS1 and PS2. The conformational diversity of γ-secretase holding PS1 (30) indicated that PS is a rather dynamic enzyme. Molecular modeling indeed suggested that PS exists in at least two different conformational states distinguished by the distance between both catalytic aspartates located on TMD6 and TMD7, respectively (31, 32).

Prior to cleavage, a substrate needs to be recognized by the enzyme which is followed by its translocation to the active site aspartates located on TMDs 6 and 7 of PS. The active site contains water required for proteolysis and thus needs to be shielded from the unpolar membrane environment. One of the current challenges in understanding the functional architecture of γ-secretase is to elucidate how its different domains cooperate in recognizing, engulfing, and unfolding of the substrate, thus preparing it for the various cleavage events (discussed in: (33)).

Here, we compared several measures of cleavage activity exhibited by γ-secretases harboring PS1 or PS2 in cell-based and cell-free assays. While confirming a lower endoproteolytic activity of PS2 relative to PS1, we also detected a lower Aβ38/Aβ37 ratio of PS2-containing *versus* PS1-containing γ-secretase. We found that the nonconserved amino acids responsible for these differences mainly reside within the transmembrane part of the PS NTF with a minor contribution by the CTF. Further, we identified TMD3 to affect initial ε-cleavage site specificity. By contrast, the efficiency of endoproteolysis is not affected by TMD3, but highly dependent on TMD6, among other TMDs within the NTF.

## Results

The aim of this study was to probe the importance of different PS domains for various aspects of C99 cleavage. C99 cleavage by γ-secretase comprises (i) ε-cleavage efficiency, *i.e.*, the yield of the various AICD species produced by endoproteolysis at all ε-sites, (ii) ε-site specificity, *i.e.*, the relative efficiency of initial cleavage at ε-sites, and (iii) processivity, *i.e.*, the relative efficiency of exopeptidase-like proteolysis across ζ-sites to the γ-sites along both product lines, including cross-over events between the lines. Altogether, these parameters determine amounts and diversity of resulting Aβ peptides. They also define the toxicity of the resulting mixture, as toxicity mainly depends on Aβ42 content (34). Furthermore, recent studies showed the association of higher Aβ38 levels in cerebrospinal fluid with lower risk of AD-related changes (35) and suggested the cerebrospinal fluid Aβ37/Aβ42 ratio as an improved biomarker for AD development (36). Thus, these observations indicate a potentially protective role of shorter Aβ peptides. Our approach was to compare various measures of cleavage activity for the PS1 and PS2 paralogs and to identify protein domains responsible for any differences uncovered. In doing so, we hoped to obtain novel insights into the functional architecture of PS.

### PS domains shaping the relative abundance of Aβ peptides

Here, we asked which parts of PS contribute to its ability to produce the major Aβ peptides. To this end, both PS variants were expressed in human embryonic kidney 293 cells stably expressing Swedish mutant APP (HEK293/sw) within a PS-free genetic background (HEK293/sw PS1/PS2^−/−^) (37). All experiments were performed with pooled stable transfectants of a given PS variant, in order to average potential variations in the expression of individual clones. We initially compared the pattern of Aβ peptides secreted by these cells to the pattern produced by endogenous γ-secretase of HEK293/sw cells by combined immunoprecipitation and MALDI-TOF mass spectrometry (IP-MS) from conditioned media. Expression of PS1 results in a pattern comprising a dominant Aβ40 peptide, minor Aβ37 and Aβ38 peptides at similar amounts, plus less prevalent Aβ39 and Aβ42; this is collectively designated here as “PS1 phenotype”. After transfection with PS2, we mainly noted a lower Aβ38/Aβ37 ratio, the “PS2 phenotype” (Fig. S1). The HEK293/sw cells expressing endogenous PS1 and PS2 produced similar ratios of Aβ including an Aβ38/Aβ37 ratio close to unity. This suggests not only that heterologously expressed PSs are principally comparable in their C99 cleavage activities to their endogenous counterparts. It also indicates that endogenous PS1 is mainly responsible for the mixture of secreted Aβ peptides by HEK293/sw cells.

**Figure 1:**
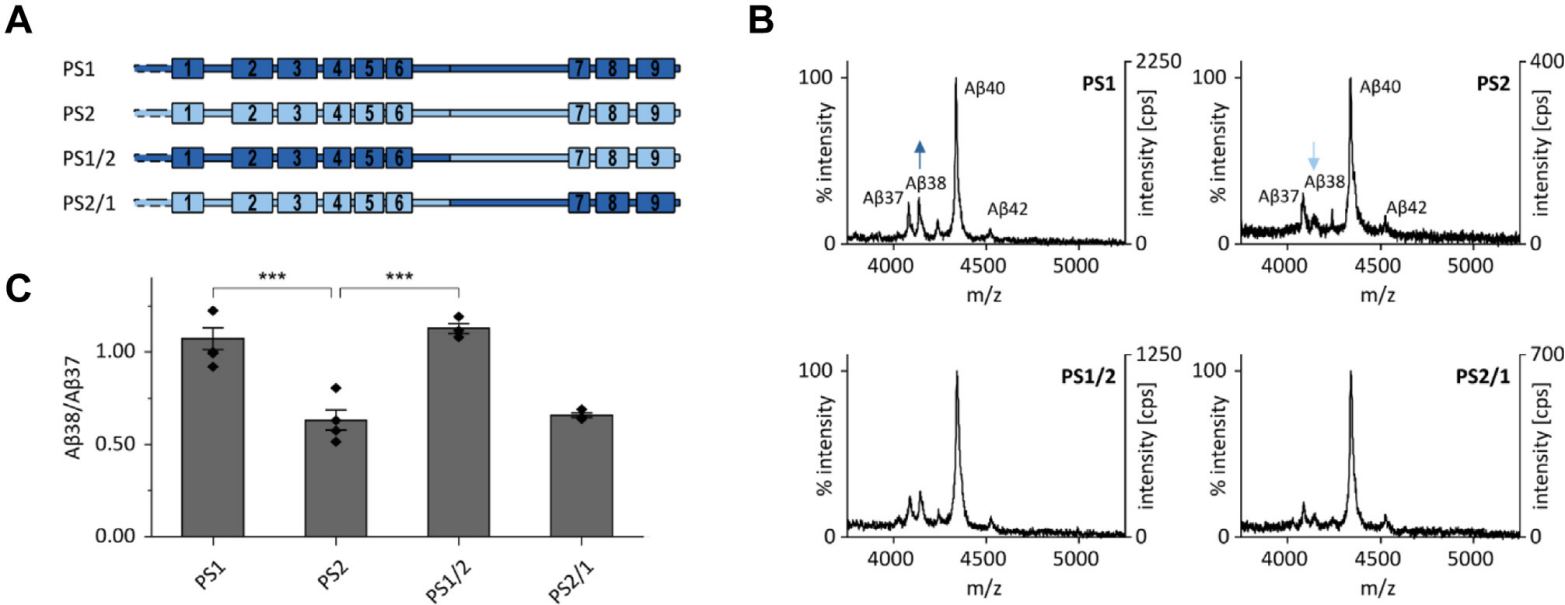
**The presenilin NTF determines differential Aβ37 and Aβ38 production by PS1 and PS2.***A*, schematic representation of the constructs. PS1 and PS2 are depicted in *dark blue* or *light blue*, respectively. *B*, exemplary total Aβ contents of conditioned media as analyzed by MALDI-TOF mass spectrometry after immunoprecipitation with antibody 4G8. The intensities of the highest peaks were set to 100% and the counts per second (cps) are given. *Arrows* mark differences in Aβ38 peak intensity. *C*, Aβ38/Aβ37 ratios change upon substitution of the presenilin NTF. Ratios were calculated from the respective peak intensities from IP-MS analyses, as shown in part (*B*). Data in (*C*) represent means ± SEM, n = 3 to 4. Individual replicates are derived from conditioned media collected from independent cultures from our pools of stably transfected cells. *Asterisks* indicate significant differences (one-way ANOVA with Dunnett’s posttest) relative to PS2 (∗∗∗*p* < 0.001). Aβ, amyloid-β; IP-MS, combined immunoprecipitation and MALDI-TOF mass spectrometry; NTF, N-terminal fragment; PS, presenilin.

Residues that are not conserved between PS1 and PS2 are distributed over the entire sequence (Fig. S2). In the following, we probed the importance of different PS domains for producing the different Aβ38/Aβ37 ratios of PS1 and PS2 by testing a range of chimeric constructs (Fig. 1*A* and Table S1) by fusing the PS NTF and CTF at the site of endoproteolysis (38, 39, 40). The exemplary mass spectra of Aβ peptides (Fig. 1*B* and Table S2) reveal that the PS1 phenotype results after fusing the complete PS1 NTF to the CTF of PS2, as in construct PS1/2. By contrast, fusing the PS2 NTF to the PS1 CTF in PS2/1 retains the PS2 phenotype. Quantifying the mean Aβ38/Aβ37 ratios from the peak heights of mass spectra from multiple samples confirms this picture (Fig. 1*C*).

**Figure 2:**
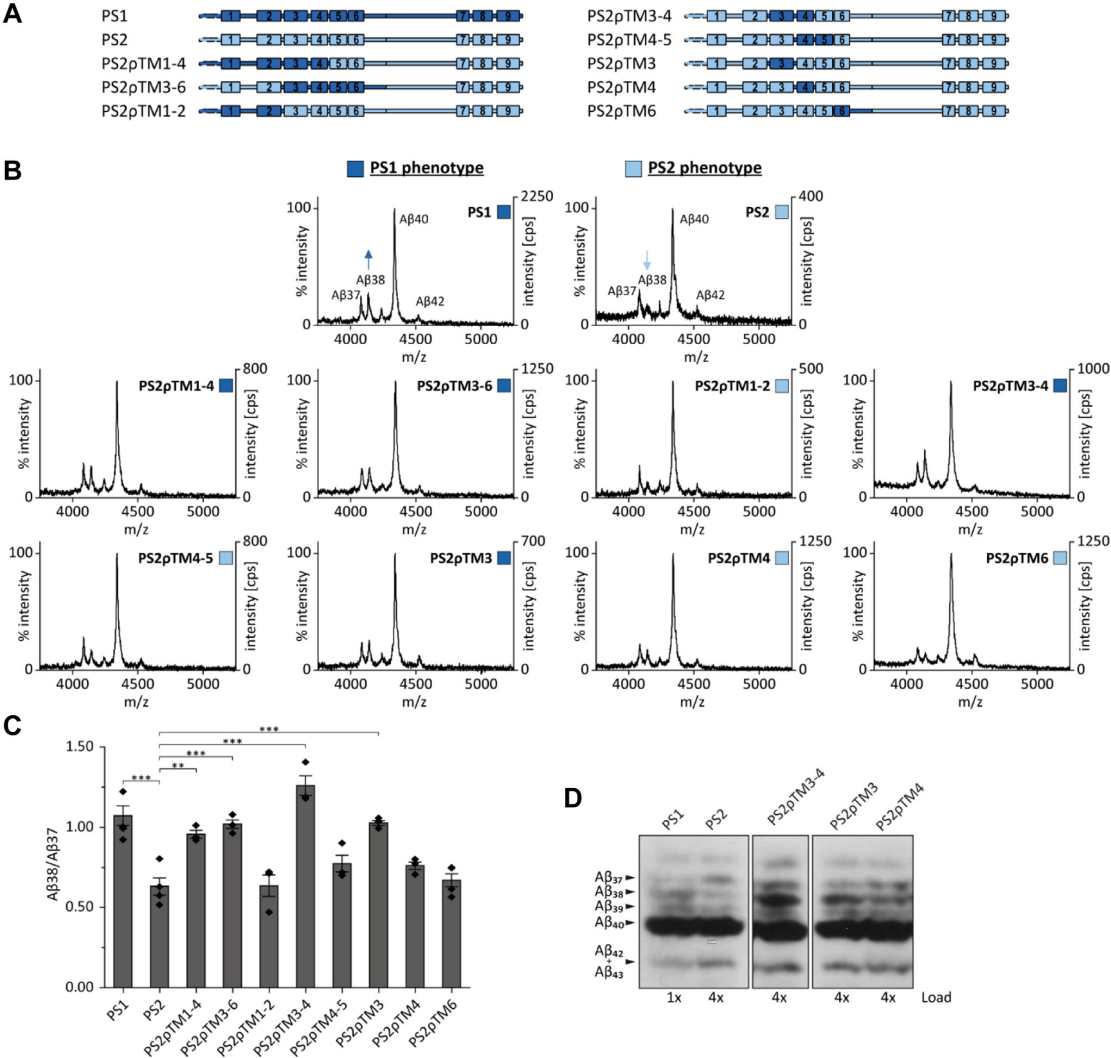
**The substitution of subdomains can phenocopy the Aβ38/Aβ37 ratio typical of PS1.***A*, schematic representation of the constructs. *B*, exemplary total Aβ contents of conditioned media were analyzed by MALDI-TOF mass spectrometry after immunoprecipitation with antibody 4G8. The spectra are categorized in ‘PS1 phenotype’ and ‘PS2 phenotype’. Data for PS1 and PS2 are reproduced from Figure. 1. *C*, Aβ38/Aβ37 ratios were calculated from the respective peak intensities from IP-MS analyses (means ± SEM, n = 3–4). Individual replicates are derived from conditioned media collected from independent cultures from our pools of stably transfected cells. *Asterisks* indicate significant differences (one-way ANOVA with Dunnett’s post-test) relative to PS2 (∗∗*p* < 0.01, ∗∗∗*p* < 0.001). We note that substituting TMD3 has the most salient effect of the single TMD substitutions tested here and that the combination of TMDs 3 and 4 in PS2ρTM3-4 has an even stronger effect than TMD3 alone. *D*, immunoprecipitation of secreted Aβ followed by separation by Tris-Bicine urea SDS-PAGE and immunoblotting confirmed the impact of TMD3 and TMD4 on Aβ generation. Aβ, amyloid-β; IP-MS, combined immunoprecipitation and MALDI-TOF mass spectrometry; PS, presenilin; TMD, transmembrane domain.

In order to map the nonconserved residues being responsible for the differential Aβ production of PS1 *versus* PS2 more finely, we next examined the importance of NTF subdomains by grafting groups of TMDs from PS1 onto the PS2 template (Fig. 2*A*). Both, PS2ρTM1-4 and PS2ρTM3-6 clearly confer a PS1 phenotype, prompting an even more granular mapping of the responsible TMDs. While PS2ρTM1-2 and PS2ρTM4-5 retain the PS2 phenotype, PS2ρTM3-4 is equivalent to PS1 (Fig. 2, *B* and *C*). At the level of individual TMDs, we find that PS2ρTM3 indeed behaves like PS1, while PS2ρTM4 and PS2ρTM6 exhibit the PS2 phenotype (Fig. 2, *B* and *C*). In a technically different approach, immunoprecipitated Aβ peptides from conditioned media were analyzed by immunoblotting after gel-electrophoretic separation using high-resolution Tris-Bicine-Urea SDS-PAGE (41). In line with the mass spectra, PS1- and PS2-containing γ-secretases show distinct production of Aβ37 and Aβ38. While the amount of Aβ38 produced by PS1-containing γ-secretase even exceeds the amount of Aβ37 (Fig. 2*D*), the inverse is true for PS2 γ-secretase. Focusing on the most relevant chimeric PS constructs described above, immunoblotting confirms a PS1 phenotype for PS2ρTM3-4 and PS2ρTM3. PS2ρTM4 appears to display similar levels of Aβ37 and Aβ38 on the immunoblot (IB) (Fig. 2*D*).

**Figure 3:**
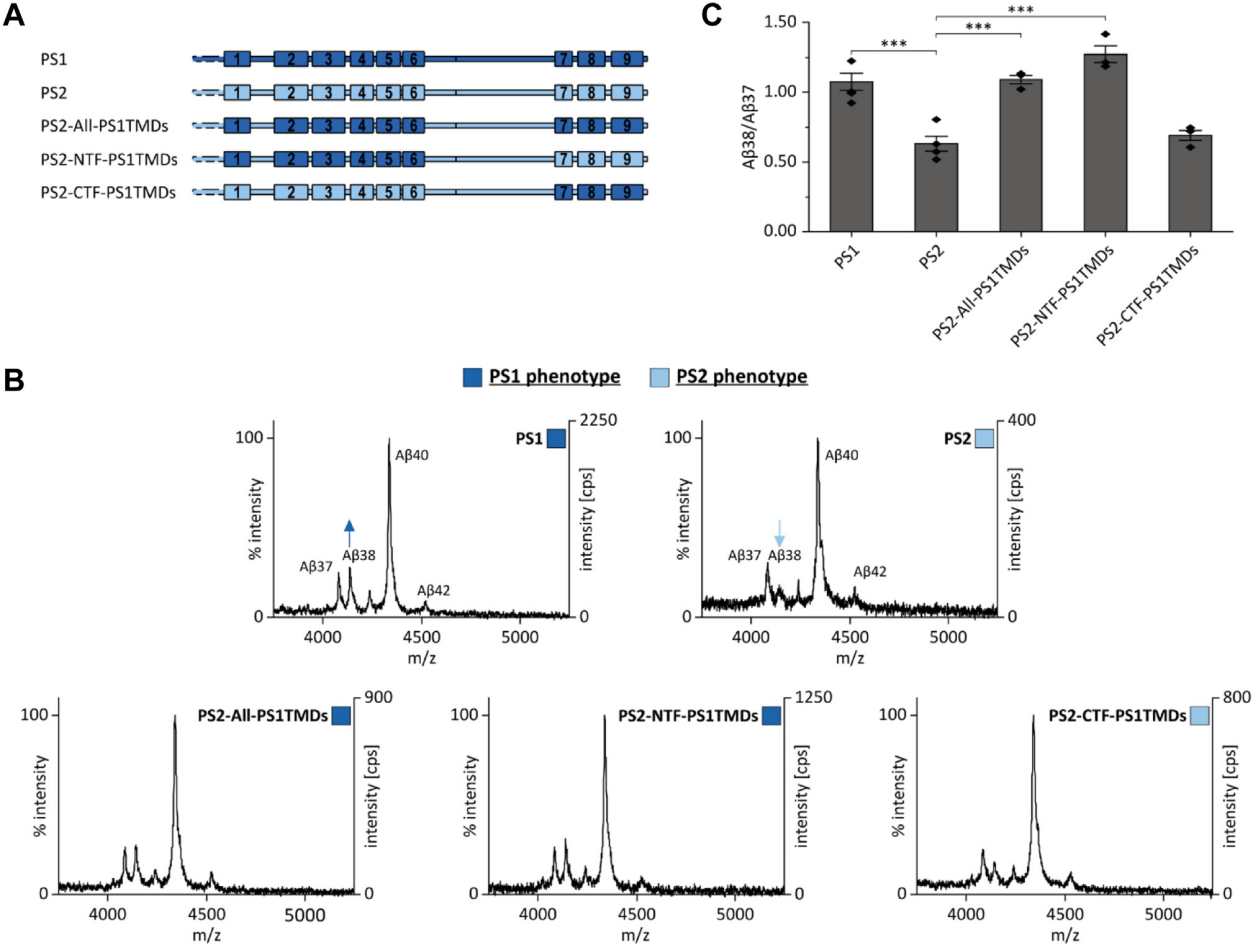
**The membrane-spanning domains of presenilin are responsible for differential Aβ38 production.***A*, schematic representation of the transfected constructs. *B*, exemplary total Aβ contents of conditioned media were analyzed by MALDI-TOF mass spectrometry after immunoprecipitation with antibody 4G8. Data for PS1 and PS2 are reproduced from Figure 1. *C*, Aβ38/Aβ37 ratios were calculated from the respective peak intensities from IP-MS analyses (means ± SEM of n = 3–4). Individual replicates are derived from conditioned media collected from independent cultures from our pools of stably transfected cells. *Asterisks* indicate significant differences (one way ANOVA with Dunnett’s post-test) relative to PS2 (∗∗∗*p* < 0.001). Aβ, amyloid-β; IP-MS, combined immunoprecipitation and MALDI-TOF mass spectrometry; PS, presenilin.

In designing the chimera presented above, we transferred the nonconserved residues of a given TMD plus both adjoining solvent-exposed loop regions; in case of TMD6, we included the C-terminal region up to the endoproteolytic cleavage site (Table S1). To collectively assess the contribution of membrane-embedded *versus* loop residues in accounting for PS1/PS2 differences, we expressed a construct where we had transferred all TMDs from PS1 to PS2 while maintaining the loop regions (Fig. 3*A*). Indeed, a construct holding all membrane-embedded amino acids from PS1 and loop residues from PS2 retains the PS1 phenotype. Further, the dominance of the NTF is preserved upon grafting only the nonconserved membrane-embedded NTF residues onto PS2 while grafting the TMDs of the CTF retains the PS2 phenotype (Fig. 3, *B* and *C*).

**Figure 4:**
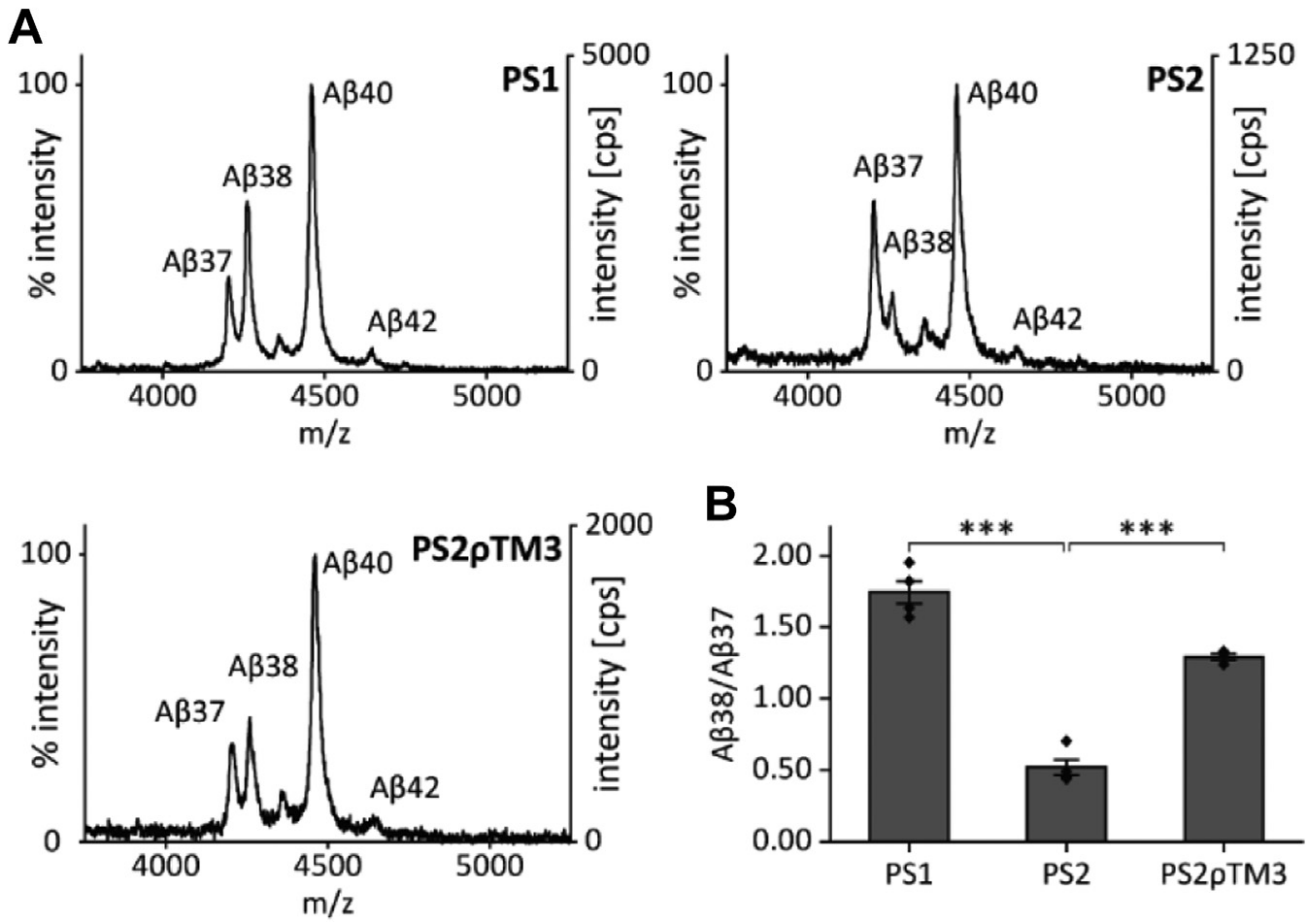
**Influence of presenilin TMD3 on Aβ production in the cell-free assay.***A*, exemplary MALDI mass spectra of Aβ peptides generated in the cell-free assay in CHAPSO-solubilized membrane fractions from cells expressing the different γ-secretase complexes and recombinant C100-His_6_ as a substrate. Subsequent to immunoprecipitation with antibody 4G8 (final concentration 4 μg/ml), total Aβ was analyzed by MALDI-TOF mass spectrometry. The intensities of the highest peaks were set to 100%. *B*, Aβ38/Aβ37 ratios were calculated from the respective peak intensities from IP-MS analyses (means ± SEM, n = 3–4). Replicates originate from individual CHAPSO-solubilized membrane fractions from independent cultures of our pooled stable transfectants. *Asterisks* indicate significant differences (one-way ANOVA with Dunnett’s post-test) relative to PS2 (∗∗∗*p* < 0.001). Aβ, amyloid-β; IP-MS, combined immunoprecipitation and MALDI-TOF mass spectrometry; PS, presenilin; TMD, transmembrane domain.

We concluded this set of experiments by asking whether the pattern of secreted Aβ peptides produced in cell-based assays (Figure 1, Figure 2, Figure 3) is influenced by differential access of C99 to PS1-containing γ-secretase in the plasma membrane relative to PS2-containing γ-secretase residing in intracellular membranes, such as endosomes (22, 23). We thus decided to compare the secreted Aβ pattern to the pattern produced in cell-free assays after membrane solubilization of γ-secretase with the detergent CHAPSO (42) by MALDI-TOF mass spectrometric analysis. In detergent, both PS paralogs are expected to have equal substrate access. As a substrate, we used the recombinant C99-based C100-His_6_ construct (17). Our results show that the Aβ38/Aβ37 ratio produced by PS1 in detergent is even higher than that in cell-based assays while the inverse is true for PS2 (Fig. 4, *A* and *B*). Also, Aβ37 and Aβ38 obtained from cell-free assays are more abundant relative to Aβ40 than in conditioned media. Given the more pronounced Aβ38/Aβ37 discrimination of the PS1 and PS2 phenotypes under cell-free conditions, we further wanted to characterize PS2ρTM3 which behaves like PS1 in the cell-based assays. Again, PS2ρTM3 exhibits an Aβ38/Aβ37 ratio that is close to that produced by PS1, however differs to both wildtype (wt) PSs. Parallel measurements of control experiments using γ-secretase inhibitor LY-411575 (43) which was shown to equally inhibit PS1 and PS2 (27) assured the observed peaks to be γ-secretase-specific (Fig. S3).

Taken together, PS2 produces a lower Aβ38/Aβ37 ratio than PS1. These data confirm and extend recent observations of differential Aβ production by PS1- and PS2-containing γ-secretases (44). They also show that the PS NTF specifies the different phenotypes. Importantly, PS1 TMD3 and to some extent PS1 TMD4 appear to confer a PS1-like phenotype when expressed in the structural context of the PS2 template. The phenotypic differences between PS1 and PS2 appear to be similar in cell-based and cell-free assays, although exopeptidase-like proteolysis leading to Aβ37 and Aβ38 relative to Aβ40 appears to be more efficient in detergent than in the natural membrane environment.

### PS domains determining ε-site specificity

Next, we asked whether the different Aβ38/Aβ37 ratios produced by the PS paralogs can be traced back to differential ε-cleavage at the origin of C99 proteolysis. Since Aβ48 and Aβ49 peptides are successively converted to shorter Aβ peptides and difficult to detect (8), we examined the corresponding AICD species ε49 and ε48. AICD produced in the cytoplasm of a cell is rapidly degraded, however, and therefore also difficult to detect (45). Thus, we monitored AICD in cell-free assays, having demonstrated a similar behavior of both PSs in cell-free and cell-based assays.

**Figure 5:**
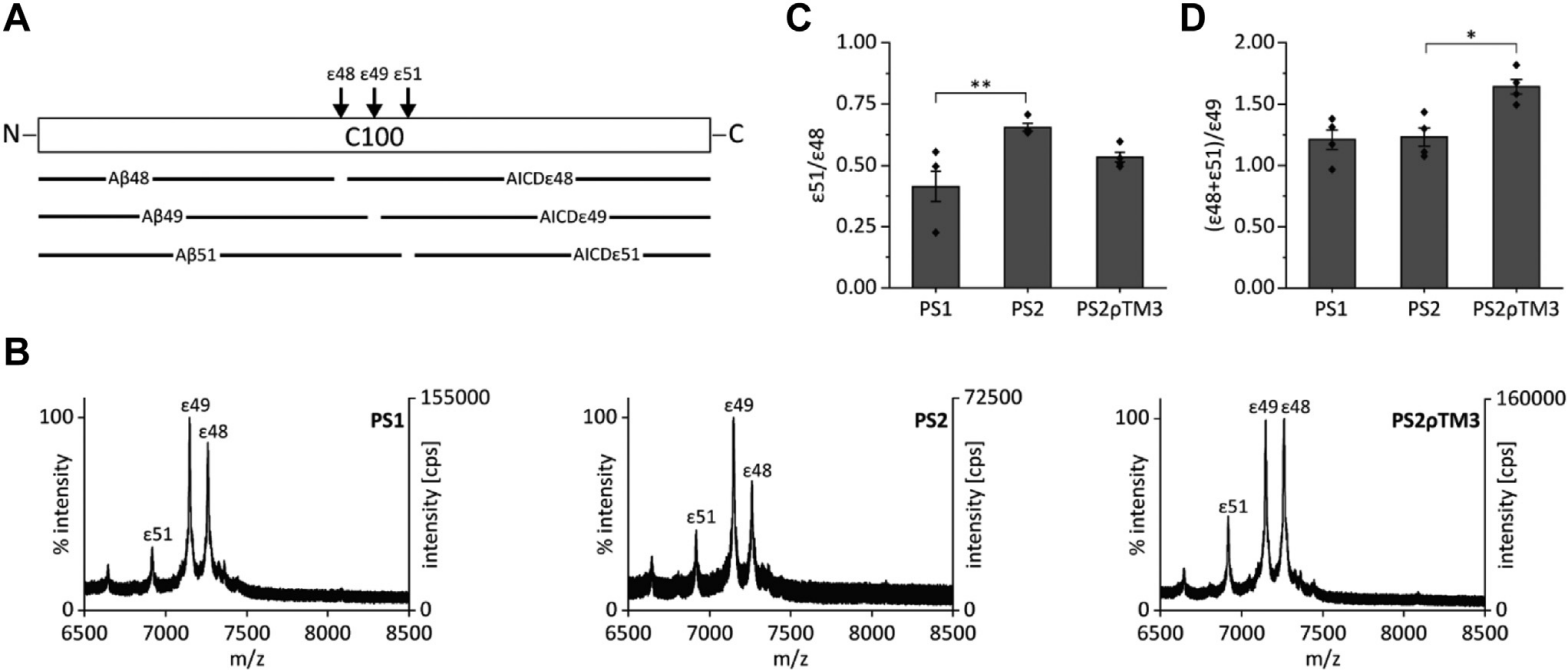
**Influence of presenilin TMD3 on the ε-cleavage specificity of γ-secretase.***A*, the Aβ and AICD products originating from cleavage at ε48, ε49, and ε51 are displayed schematically. *B*, exemplary MALDI mass spectra of AICD peptides generated in the cell-free assays. Total AICD from the reaction volumes was analyzed by MALDI-TOF mass spectrometry subsequent to immunoprecipitation with antibody Y188. The spectra were recorded with an instrument different from that used for Figure 1, Figure 2, Figure 3, Figure 4, resulting in higher overall cps. *C* and *D*, peak heights were quantified, and ratios calculated to investigate preferences for either (*C*), the initial cleavage site within the Aβ42 product line or for (*D*), the Aβ40 *versus* Aβ42 product lines. Data in (*C*) and (*D*) represent means ± SEM, n = 4. Replicates originate from individual CHAPSO-solubilized membrane fractions from independent cultures of our pooled stable transfectants. *Asterisks* indicate significant differences (one-way ANOVA with Dunnett’s post-test) relative to PS2 (∗*p* < 0.05, ∗∗*p* < 0.01)). Aβ, amyloid-β; AICD, APP intracellular domain; PS, presenilin; TMD, transmembrane domain.

AICD peptides ε48 (51 residues), ε49 (50 residues), and ε51 (48 residues) result from cleavages at ε48-, ε49-, and ε51-sites, respectively (Fig. 5*A*). Assessing these AICD peptides in a detergent-solubilizate showed that PS2 produced more AICDε51 than PS1, at the expense of AICDε48 (Fig. 5*B*). This manifests itself in a higher mean ε51/ε48 ratio exhibited by PS2 relative to PS1 (Fig. 5*C*). At the same time, the (ε48+ε51)/ε49 ratios produced by both PS paralogs are indistinguishable (Fig. 5*D*). Since cleavages at both the ε48- and the ε51-sites result in the Aβ42 product line (9), both PSs enter the Aβ40 and Aβ42 product lines with similar efficiency although initial PS2 cleavage is partially shifted from ε48 to ε51. Controls with γ-secretase inhibitor LY-411575 showed the analyzed peaks to be γ-secretase-specific (Fig. S3). To investigate whether the PS1-like Aβ production by PS2ρTM3 (Fig. 2) originates from different ε-site preferences, we investigated initial cleavage by this chimera. In terms of the AICD ε51/ε48 ratio, PS2ρTM3 ranges in between PS1 and PS2 (Fig. 5*C*). Also, relative to AICDε49, PS2ρTM3 produced more AICDε48 than PS1 and more AICDε51 than PS2 (Fig. 5*B*). Accordingly, the (ε48+ε51)/ε49 ratio of PS2ρTM3 significantly exceeds the corresponding ratios generated by PS1 or PS2 (Fig. 5*D*).

Taken together, the partial shift of initial cleavage from the ε48-site to the ε51-site by PS2 does not affect the efficacy by which the PS paralogs enter both product lines. This higher preference of PS2 for the ε51-site seems at least partially determined by TMD3. In addition, the PS1 TMD3 within the PS2 framework enhances ε48 and ε51 cleavages relative to both wt PSs.

### PS domains defining ε-cleavage efficiency

**Figure 6:**
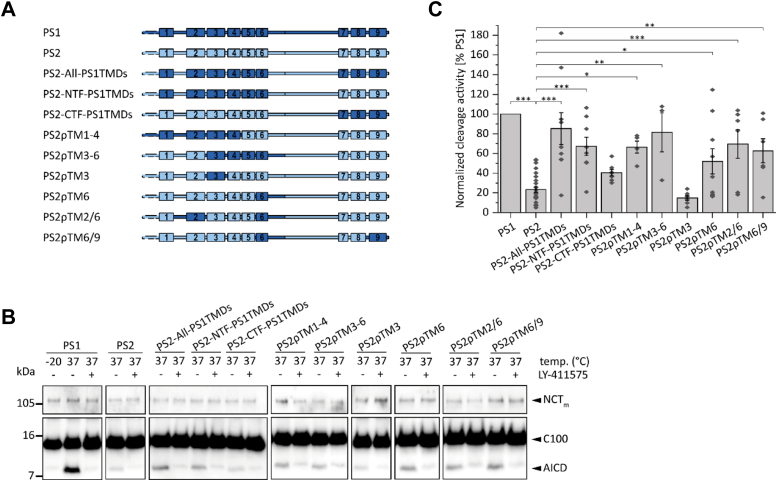
**Relevance of presenilin domains for the efficiency of ε-cleavage.***A*, schematic representation of the constructs. *B*, representative immunoblots used for AICD and NCT_m_ quantification from cell-free assays. The cleavage efficiency of recombinant C100-His_6_ substrate was analyzed in CHAPSO-solubilized membrane fraction containing the different γ-secretase complexes. Signal intensities of generated total AICD were measured on immunoblots. *C*, signal intensities were first normalized to signals of NCT_m_ and are expressed relative to the level seen with PS1. Appropriate maturation of the respective γ-secretase complexes was verified prior to analysis (see: Fig. S4). Data in (*C*) represent means ± SEM, n ≥ 3. *Asterisks* indicate significant differences (one-way ANOVA with Dunnett’s post-test) relative to PS2 (∗*p* < 0.05, ∗∗*p* < 0.01, ∗∗∗*p* < 0.001). AICD, APP intracellular domain; PS, presenilin; TMD, transmembrane domain.

In this set of experiments, we compared the efficiency by which γ-secretases holding PS1 or PS2 perform ε-cleavage and tested various chimeric constructs in order to delineate individual domains accounting for differences between both isoforms (Fig. 6*A*). To this end, we determined total AICD levels obtained after cleavage in CHAPSO-solubilized membranes by immunoblotting (Fig. 6*B*) and, following quantitation, expressed AICD production by PS2 and chimeric constructs relative to PS1 (Fig. 6*C*). Figure 6 reveals that PS2 γ-secretase produces only 23% AICD of PS1 γ-secretase, thus confirming previous reports having indicated a higher PS1 activity (22). In assessing the importance of the PS NTF *versus* its CTF, we employed a gain-of-function approach by using the weaker PS2 as a template onto which PS1 domains were grafted (Fig. 6*A*). This minimizes potential pleiotropic effects of altered primary structure that are often encountered when studying loss-of-function after deleting domains or fusing them to unrelated proteins. We first compared the impact of transferring all TMDs from PS1 to PS2 while maintaining the solvent-exposed loops. AICD production by the PS2-All-PS1TMDs chimera showed 85% of PS1 activity (Fig. 6*C*), thus revealing the importance of the TMDs for the efficiency of cleavage. ε-Cleavage efficiency is mostly determined by the membrane-embedded amino acids of the NTF (PS2-NTF-PS1TMDs: 67% of wt PS1), while the respective CTF residues had a smaller share (PS2-CTF-PS1TMDs: 40% of wt PS1).

For a more detailed functional mapping of the PS NTF found to dominate ε-efficiency, we scrutinized several of the chimeric constructs presented above where subdomains of the PS1 NTF are grafted onto the PS2 template. Thereby, we found PS2ρTM1-4 and PS2ρTM3-6 to exhibit 67% or 81% of PS1 efficiency, respectively. Interestingly, PS2ρTM3 produced only 15% AICD, while PS2ρTM6 yielded an impressive 52% AICD (Fig. 6*C*). Since PS2ρTM3 appears not to account for the increased activity of PS2ρTM1-4, we assessed a potential influence of PS TMD2 on ε-cleavage efficiency. TMD2 in combination with TMD6 was previously proposed to be involved in substrate entry (32, 33, 46). To test the importance of this putative entry site for PS ε-cleavage efficiency, we combined PS1 TMD2 and TMD6 in PS2ρTM2/6 (Fig. 6*A*). Substrates had also been proposed to enter between TMD6 and TMD9 (47, 48) prompting us to examine a PS2ρTM6/9 construct. However, neither PS2ρTM2/9 nor PS2ρTM6/9 showed ε-cleavage efficiencies above that of PS2ρTM6 with its single TMD substitution (Fig. 6*C*).

To control for maturation and concentration of the different γ-secretase complexes, we visualized their subunits by immunoblotting: fully glycosylated NCT_m_, as indicated by its higher molecular mass relative to core-glycosylated NCT, the PS NTFs and CTFs as an indicator of autoproteolytic cleavage, and PEN-2 (Fig. S4).

Taken together, our results reveal domains that specify the difference in PS1/PS2 cleavage efficiency. The nonhomologous membrane-embedded residues of the NTF are more relevant than those of the CTF, and TMD6 makes a strong individual contribution.

## Discussion

The experimental approach outlined here exploits functional differences between PS1 and PS2 in order to identify protein domains governing various aspects of substrate processing, like substrate binding, engulfing, and cleavage. We reasoned that investigating chimera of those highly homologous proteins may cause fewer undesired pleiotropic effects on their structure than truncations, deletions, or fusions with sequences from unrelated proteins. A limitation of our approach is that domains that determine functional properties shared by both paralogs may not be identified. We further acknowledge that the type of expressing cells might affect substrate processing by PS. However, with respect to APP processing, HEK293/sw cells produce Aβ species in ratios resembling those observed in brain-derived cells (*e.g.*, (49, 50, 51)). Two major differences between γ-secretase complexes containing PS1 or PS2 form the basis of our strategy. First, γ-secretase complexes holding PS1 or PS2 exhibit a remarkably different sequence-specificity of C99 processing, that is, a reduced production of Aβ38 by PS2 compared to PS1 being paralleled by an increase in Aβ37. While different Aβ38 levels have recently been reported (28, 29), differential Aβ37 production has not been described before. Although low signal intensities of Aβ42 prevented us to calculate reliable Aβ42/Aβ40 ratios from IP-MS measurements, qualitatively, Aβ42 levels relative to Aβ40 appeared to be similar for PS1 and PS2. This is in line with studies reporting a similar Aβ42/Aβ40 ratio for both PS paralogs (22, 23) but inconsistent with other studies describing a higher Aβ42/Aβ40 ratio for PS2-containing γ-secretase (52, 53). For both paralogs, ε49 appears to be the major cleavage site, followed by ε48, which agrees with previous reports (28, 54, 55). Interestingly, we show that PS2 uses the ε51-site more frequently than PS1 and does so at the expense of ε48. To our knowledge, this difference in ε-cleavage has not been shown before. Rather, the ε-cleavage specificity had been reported to be similar for PS1 and PS2 using the less sensitive IB analysis (29). Moreover, AICDε51 had not been determined in some previous studies (28, 29). Our data are consistent, however, with previously reported AICD production by HeLa cells expressing both PS paralogs. There, the plasma membrane generated almost exclusively AICDε49, while endosomes produced mostly AICDε51 (56). Since PS2-containing γ-secretase mostly resides in late endosomes and lysosomes (22, 23), AICDε51 production most likely had originated from PS2, in line with our current results. Since Aβ51 is first processed to Aβ48, it also enters the Aβ42 product line (9, 10, 57). Altogether, PS1 and PS2 thus initiate the Aβ42 line with a slightly higher efficiency than the Aβ40 line (Fig. 5*D*). In turn, this suggests that it is the downstream processing of the Aβ peptides that leads to the observed differences in Aβ38/Aβ37 ratio between PS1 and PS2.

**Figure 7:**
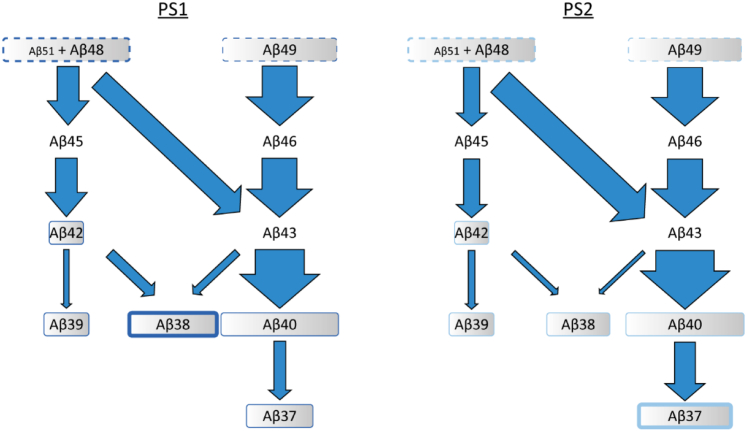
**Product line usage of PS1 and PS2.** The efficiencies of ε-cleavage as well as of proteolysis along both product lines and crossover events between them, as indicated by previous data (9, 10) and our current results, can explain the differential Aβ38/Aβ37 ratios of PS1 and PS2. The width of the boxes containing the peptide species represents the relative amount of peptide at the start (*dashed lines*) or at the end (*solid lines*) of the respective Aβ product line. *Arrow* width indicates the presumed efficiency of respective cleavage steps. In particular, the more frequent use of the Aβ48→Aβ43 transition might explain the higher Aβ37 production by PS2. Aβ, amyloid-β; PS, presenilin.

At which stage then does Aβ trimming differ between both PSs to produce the lower Aβ38/Aβ37 ratio exhibited by PS2? Assuming that Aβ42 derives from Aβ48, one would expect Aβ42 to be the major Aβ species. However, consistent with previous studies (22, 25, 28) both, PS1- and PS2-containing γ-secretases produce Aβ40 as the major species, thus suggesting frequent crossover from the Aβ42 product line to the Aβ40 line. To date, the generation of Aβ43 from its precursor Aβ48 is the sole reported crossover point that could allow for this product line switch (9, 10) (Fig. 7). Indeed, PS2 might use the Aβ48→Aβ43 switch more frequently than PS1, as Aβ43 generates Aβ37 *via* Aβ40. Alternatively, PS2 might be more efficient in a previously reported rare direct conversion of Aβ42 to Aβ37 (9) or use additional, so far unidentified, crossover points.

Second, γ-secretase holding PS2 is much less efficient in AICD production than PS1-containing γ-secretase. This was shown by our experiments on cleavage efficiency with detergent-solubilized membranes as enzyme source where both types of γ-secretase are expected to have equal access to the substrate. This confirms previous studies where AICD production by γ-secretase holding PS2 relative to PS1 was somewhat reduced *in cellulo* using a luciferase-based reporter assay conducted on BD8 cells (58) or strongly diminished in a cell-free assay (22, 29).

Analyzing PS1/PS2 chimeric enzymes shed light on the structural domains defining both, the specificity and efficiency of C99 cleavage. Cleavage specificity, as expressed by the Aβ38/Aβ37 ratio, is mainly accounted for by the NTF. Furthermore, we found the membrane-embedded nonconserved residues to be mainly responsible for this paralog-specific phenotype. The same holds true for the efficiency of AICD production. By implication, the specificity of Aβ37 and Aβ38 production as well as the efficiency of ε-cleavage appear both to be mainly mediated by interactions of the substrate with TMDs of the NTF of PS. That the NTF is responsible for paralog-specific ε-cleavage efficiency had previously been observed in a cell-based assay, albeit without statistical significance (58).

At a more granular structural level, the single TMD exchange resulting in chimera PS2ρTM3 produced a PS1-like Aβ38/Aβ37 ratio. How does TMD3 affect Aβ production? On the one hand, one straightforward explanation would imply a role of TMD3 in Aβ trimming after ε-cleavage. After all, the specificity of ε-cleavage is not the root cause of Aβ38/Aβ37 ratios differing between wt PS1 and PS2, as noted above (Fig. 5*D*). On the other hand, we found that exchanging TMD3 had profound effects on the specificity of ε-cleavage since PS2ρTM3 partially mimics PS1 in its ε51/ε48 ratio (Fig. 5*C*) albeit this effect is unlikely to affect the Aβ38/Aβ37 ratio. In addition, PS2ρTM3 produced higher levels of AICDε48 plus AICDε51 relative to AICDε49 than both wt PSs (Fig. 5*D*). Although this effect may partially explain an elevated Aβ38 level resulting from a favored entry into the Aβ42 product line (Fig. 7) (9), it is not suited to explain the elevated Aβ38/Aβ37 ratio of PS1 compared to PS2. In other words, TMD3 clearly influences the choice of initial cleavage site; however, the extent to which this effect and an effect of TMD3 on subsequent trimming define the differential Aβ38/Aβ37 ratios of PS1 *versus* PS2 is currently unknown. Along this line, it may be worth noting that the Aβ38/Aβ37 ratio produced by PS2ρTM3-4 exceeds that of PS2ρTM3. Conceivably, therefore, TMD3 may cooperate with TMD4. That TMD3 affects Aβ production is supported by previous domain-swapping experiments where TMD3 was the only domain to alter Aβ42 ratios, whereas mutation of other TMDs completely abolished substrate cleavage (59). It is likely that additional structural determinants of Aβ production may be identified in the future.

How might the different TMDs mechanistically exert their influence? In general, the functional dominance of membrane-embedded over loop residues, as found in this study, is not surprising as the formers dictate the noncovalent interactions between the TM helices and thus the structure and dynamics of a membrane protein (60). Indeed, simulations suggest correlated motions of TMDs being responsible for transitions between various conformational states of PS (61). Specifically, a range of studies have elucidated the participation of TMD3 in the formation of the catalytic pore and of the active site in PS (62). The structure of the substrate/enzyme complex has been determined by cryogenic electron microscopy (cryo-EM). In this structure, the C83 substrate is located between TMD2 and TMD3 of PS1 with apparent interactions between W165 of PS1 TMD3 and V44 of C83 as well as between S169 of the PS1 TMD3 and I41 of C83 (63). While these C83-contacting residues are conserved, other TMD3 residues vary between both PSs, possibly affecting the precise positioning of the substrate TMD which determines ε-specificity. That TMD3 contributes to formation of an aqueous catalytic pore was initially suggested by a cysteine labeling approach (64). Follow-up studies using the γ-secretase modulator E2012 further indicated its involvement in Aβ trimming. Binding of E2012 induces conformational changes of TMD3 resulting in an expansion of the catalytic cavity which was reported crucial for Aβ42 reduction (65). Furthermore, the importance of TMD3 for Aβ production is underlined by the fact that 13% of all familial AD mutations with assumed and confirmed pathogenicity are located in TMD3 (https://www.alzforum.org/mutations) albeit this TMD comprises less than 7% of all PS1 amino acids. Among these is the very aggressive L166P mutation, which is associated with an early onset of AD (66). Photoaffinity mapping showed L166P to site specifically alter the efficiency of crosslinking between the PS1 NTF and the ε-sites of C99 (67, 68), indicating that TMD3 functionally interacts with the initial cleavage region. Further, TMD3 contacts TMD4 which itself does not contact the substrate in the cryo-EM structure (30, 63). Notably however, a residue near the C-terminus of TMD4 can be crosslinked to residues 383 or 387 bordering the catalytic D385 of TMD7 (69). Thus, TMD4 appears to visit the catalytic site at least transiently, which may explain its apparent cooperation with TMD3 in our current study.

Surprisingly, TMD3 appears to have no discernible impact on ε-cleavage efficiency as indicated by the PS2-like AICD production exhibited by PS2ρTM3 in cell-based assays. It follows that the final substrate positioning that is thought to govern ε-site specificity is uncoupled from the efficiency of ε-cleavage. Rather, it is likely that an earlier step, such as the ease of substrate translocation toward the catalytic site is crucial for ε-efficiency. Various lines of evidence had previously suggested a role of PS TMDs 2, 6, and 9 in substrate recognition (reviewed in: (33, 70)). More specifically, entry gates between TMDs 2 and 3 (71), TMDs 2 and 6 (32, 46), and TMDs 6 and 9 (47, 48) had been proposed. TMD6 might be a particularly crucial site of initial substrate binding. Exchanging either part of TMD6 or TMD2 of PS for nonrelated TMDs had abolished labeling by a photoprobe based on a peptidic substrate mimic (59). In a comparison of different cryo-EM structures, TMD6 showed the highest tilt angle variations of all TMDs that correlated to the distance between both catalytic aspartates (72). As a small distance between these aspartates is required for cleavage, TMD6 dynamics might explain the ε-cleavage efficiency of PS2ρTM6 which approached that of PS1. Unexpectedly, combining PS1 TMD6 with TMD2 or TMD9 did not further increase ε-efficiency. This result does not challenge a role of TMDs 2, 6, and 9 in substrate recognition. Rather, it is consistent with the idea that TMD2 and TMD9 do not define the differences in PS1 and PS2 activity. It is currently not clear how the PS1 TMDs within PS2ρTM1-4 account for higher-than-PS2 activity of this chimera. A previously reported TMD2/TMD3 entry site (71), a concerted movement of TMD3 and TMD1 (65), or the stabilization of TMD3 by TMD4 during cleavage (62) may help to explain the enhanced efficiency of PS2ρTM1-4. However, the exact mechanism remains elusive and needs further investigation.

We conclude that TMD3 is a strong determinant of the choice of the ε-site but not of ε-cleavage efficiency. By contrast, other TMDs, including TMD6, contribute to ε-cleavage efficiency but do not determine the Aβ38/Aβ37 ratio. Thus, TMD6 may have a major impact on substrate acquisition and/or translocation to the active site. It may be noteworthy that previous Michaelis-Menten analyses with detergent-solubilized enzyme uncovered lower K_m_ and V_max_ values of PS2 relative to PS1 (27). One aspect by which PS2 differs from PS1 may thus correspond to a higher substrate affinity, which is approximated by K_m_. Naturally, a higher affinity does not explain the lower PS2 efficiency found here and elsewhere (22, 29, 58). Another aspect distinguishing both paralogs appears to a differential substrate turnover, as indicated by the lower V_max_ of PS2. The V_max_ value is influenced by any step downstream of initial substrate binding (73), including the efficiency by which an initially bound substrate TMD is translocated toward the active site. As the nonconserved TMD6 residues are located at the interface between the TMD6 and the TMD2 helices (63), ε-cleavage efficiency may depend on the ease by which this interface transiently breaks to allow substrate translocation between TMDs 2 and 6 (32, 46). Alternatively, ε-cleavage efficiency may be governed by the contribution of TMD6 to the active site where conformational changes of TMD6 were observed after substrate binding (63, 74). Future studies on the substrate specificity of PS1 *versus* PS2 may benefit from the recently solved cryo-EM structure of PS2 (75) which, although nearly identical to that of PS1, may show differences in yet to be determined substrate-bound forms. In any case, by comparing differential C99 processing by both PSs and chimeric variants thereof, our current study helps to identify important determinants of the specificity and efficiency of cleavage.

Finally, we note that the physiological roles of PS1 and PS2 involve substrates other than C99. As with C99, differential cleavage of such other substrates by both PS paralogs has been reported and is likely to depend on their differential subcellular localizations (22, 23). For example, the late endosome- and lysosome-localized premelanosome protein and tyrosine-related protein are mostly cleaved by PS2, but barely by PS1. By contrast, N-cadherin found at the cell surface is predominantly cleaved by PS1 (23, 53).

## Experimental procedures

### Antibodies

Antibodies to the PS1 NTF (2G7 (68), IB: 1 μg/ml), the PS1 CTF (5E12 (76), IB: 2 μg/ml), the PS2 NTF (2972 (77), IB: 1:500), the PS2 CTF (BI.HF5c (78), IB: 1:2000), and total Aβ (2D8 (24), IB: 3 μg/ml and 3552 (79) immunoprecipitation (IP): 1:500) have been described previously. The antibodies N1660 to NCT (Sigma, IB: 1:5000), Penta-His (Qiagen, IB: 1:5000), Y188 to the APP C terminus (Abcam, IP: 1:2500), and 4G8 (Biolegend, IP: 2.5 μg/ml unless stated otherwise) were obtained from the indicated companies. Rabbit polyclonal antibody 8557 (IB: 1 μg/ml) was raised against residues 4 to 15 of human PEN-2.

### cDNA constructs and transfection of mammalian cells

cDNA constructs encoding the individual human PS variants, N-terminally tagged with hexahistidine sequences, were generated by Gibson Assembly (80) (New England Biolabs) and cloned into the mammalian expression vector pcDNA4/HisC (Invitrogen). The required cDNA sequences were amplified *via* standard PCR. Gibson Assembly was performed according to the manufacturer’s protocol. Table S1 lists the identities of the fused fragments. The identity of the TMDs corresponds to the respective annotations in the cryo-EM structure (pdb: 5fn3 (30)). The integrity of all cDNA constructs was verified by DNA sequencing. HEK293/sw and HEK293/sw PS1/2 ^−/−^ dKO cells (37) were stably transfected and cultured in the presence of the selection antibiotic Zeocin (InvivoGen) to ensure genomic integration as previously described (81). For the sake of averaging the expression of PS variants on substrate processing, all clones obtained for a given variant were pooled after antibiotic selection and co-cultivated.

### Protein analysis and cleavage assays

PS1, PS2, NCT, and PEN-2 were detected in cell lysates by direct immunoblotting as described (82). IP-MS analysis of Aβ peptides generated in cell-based or cell-free assays was done as described previously (76) using antibody 4G8 and the 4800 MALDI TOF/TOF Analyzer (Applied Biosystems). AICDs generated in cell-free assays were analyzed by IP-MS using antibody Y188 and MALDI-TOF mass spectrometry (rapifleX Tissuetyper, Bruker). MALDI spectra were quantitatively evaluated by determining peak heights and averages were calculated.

Individual Aβ species were also analyzed by immunoblotting, using Tris-Bicine-Urea SDS-PAGE (41) after IP with antibody 3552. For separation of Aβ species, we used a 12% stacking gel without urea and an 8% separation gel containing 8 M urea.

For cell-free γ-secretase assays, membrane fractions from HEK293/sw PS1/2^−/−^ cells co-expressing wt PS1, PS2, or chimeric PS variants were prepared as described (3) and subsequently solubilized with 1% CHAPSO [1% CHAPSO, 150 mM sodium citrate pH 6.4, 1x cOmplete protease inhibitor (Roche)]. γ-Secretase activity was assessed as described before (76) except that 1.2 μM LY-411575 (43) (Merck) was used for γ-secretase inhibition. To determine the ε-cleavage efficiency of a given γ-secretase complex, the mixture of AICDs generated in cell-free assays was separated from other proteins on Tris-Tricine gels (83) with subsequent immunoblotting using the Penta-His antibody. Quantification of signal intensities from IBs was performed using a Western Blot imager (Fusion FX, Vilber Lourmat) and the Image Studio Lite Ver. 5.2 software (LI-COR). When quantifying band intensities, we sought to minimize potential variations of AICD production that may result from different levels of functional γ-secretase. Thus, we normalized AICD staining intensity to that of the mature, fully glycosylated NCT_m_ determined in parallel.

To determine ε-cleavage specificity, the quantity of individual AICD species was investigated as described above. Likewise, Aβ peptides generated in cell-free assays were investigated as described above. Signal averages were calculated from data obtained from multiple membrane preparations.

### Statistical analysis

Data are presented as the mean value ± SEM and were analyzed using one-way analysis of variance (ANOVA) and post hoc Dunnett’s test. Statistical significance was defined as *p* < 0.05. The data were analyzed using GraphPad Prism 9 (GraphPad Software).

## Data availability

All source data required for determination of mean Aβ and AICD ratios or the cleavage efficiency will be made available upon request.

## Supporting information

This article contains supporting information (30).

## Conflict of interest

The authors declare they have no conflicts of interest with the contents of this article.

## References

[1] Guner G., Lichtenthaler S.F. (2020). The substrate repertoire of γ-secretase/presenilin. Semin. Cell Dev. Biol..

[2] Selkoe D.J., Hardy J. (2016). The amyloid hypothesis of Alzheimer's disease at 25 years. EMBO Mol. Med..

[3] Sastre M., Steiner H., Fuchs K., Capell A., Multhaup G., Condron M.M. (2001). Presenilin-dependent γ-secretase processing of b-amyloid precursor protein at a site corresponding to the S3 cleavage of Notch. EMBO Rep..

[4] Gu Y., Misonou H., Sato T., Dohmae N., Takio K., Ihara Y. (2001). Distinct intramembrane cleavage of the b-amyloid precursor protein family resembling γ-secretase-like cleavage of Notch. J. Biol. Chem..

[5] Yu C., Kim S.H., Ikeuchi T., Xu H., Gasparini L., Wang R. (2001). Characterization of a presenilin-mediated amyloid precursor protein carboxyl-terminal fragment γ. Evidence for distinct mechanisms involved in γ-secretase processing of the APP and Notch1 transmembrane domains. J. Biol. Chem..

[6] Weidemann A., Eggert S., Reinhard F.B., Vogel M., Paliga K., Baier G. (2002). A novel ε-cleavage within the transmembrane domain of the Alzheimer amyloid precursor protein demonstrates homology with notch processing. Biochemistry.

[7] Qi-Takahara Y., Morishima-Kawashima M., Tanimura Y., Dolios G., Hirotani N., Horikoshi Y. (2005). Longer forms of amyloid β protein: implications for the mechanism of intramembrane cleavage by γ-secretase. J. Neurosci..

[8] Takami M., Nagashima Y., Sano Y., Ishihara S., Morishima-Kawashima M., Funamoto S. (2009). γ-Secretase: successive tripeptide and tetrapeptide release from the transmembrane domain of β-carboxyl terminal fragment. J. Neurosci..

[9] Matsumura N., Takami M., Okochi M., Wada-Kakuda S., Fujiwara H., Tagami S. (2014). γ-Secretase associated with lipid rafts: multiple interactive pathways in the stepwise processing of β-carboxylterminal fragment. J. Biol. Chem..

[10] Olsson F., Schmidt S., Althoff V., Munter L.M., Jin S., Rosqvist S. (2014). Characterization of intermediate steps in amyloid beta (Aβ) production under near-native conditions. J. Biol. Chem..

[11] Okochi M., Tagami S., Yanagida K., Takami M., Kodama T.S., Mori K. (2013). γ-Secretase modulators and presenilin 1 mutants act differently on presenilin/γ-secretase function to cleave Aβ42 and Aβ43. Cell Rep..

[12] Lichtenthaler S.F., Haass C., Steiner H. (2011). Regulated intramembrane proteolysis - lessons from amyloid precursor protein processing. J. Neurochem..

[13] De Strooper B., Iwatsubo T., Wolfe M.S. (2012). Presenilins and γ-secretase: structure, function, and role in Alzheimer disease. Cold Spring Harb. Perspect. Med..

[14] Steiner H., Fukumori A., Tagami S., Okochi M. (2018). Making the final cut: pathogenic amyloid-β peptide generation by γ-secretase. Cell Stress.

[15] Thinakaran G., Borchelt D.R., Lee M.K., Slunt H.H., Spitzer L., Kim G. (1996). Endoproteolysis of presenilin 1 and accumulation of processed derivatives *in vivo*. Neuron.

[16] Beher D., Wrigley J.D., Nadin A., Evin G., Masters C.L., Harrison T. (2001). Pharmacological knock-down of the presenilin 1 heterodimer by a novel γ-secretase inhibitor: implications for presenilin biology. J. Biol. Chem..

[17] Edbauer D., Winkler E., Regula J.T., Pesold B., Steiner H., Haass C. (2003). Reconstitution of γ-secretase activity. Nat. Cell Biol..

[18] Fukumori A., Fluhrer R., Steiner H., Haass C. (2010). Three-amino acid spacing of presenilin endoproteolysis suggests a general stepwise cleavage of γ-secretase-mediated intramembrane proteolysis. J. Neurosci..

[19] Wolfe M.S., Xia W., Ostaszewski B.L., Diehl T.S., Kimberly W.T., Selkoe D.J. (1999). Two transmembrane aspartates in presenilin-1 required for presenilin endoproteolysis and γ-secretase activity. Nature.

[20] Langosch D., Steiner H. (2017). Substrate processing in intramembrane proteolysis by γ-secretase - the role of protein dynamics. Biol. Chem..

[21] Wolfe M.S. (2020). Substrate recognition and processing by γ-secretase. Biochim. Biophys. Acta Biomembr..

[22] Meckler X., Checler F. (2016). Presenilin 1 and presenilin 2 target γ-secretase complexes to distinct cellular compartments. J. Biol. Chem..

[23] Sannerud R., Esselens C., Ejsmont P., Mattera R., Rochin L., Tharkeshwar A.K. (2016). Restricted location of PSEN2/γ-secretase determines substrate specificity and generates an intracellular Aβ pool. Cell.

[24] Shirotani K., Tomioka M., Kremmer E., Haass C., Steiner H. (2007). Pathological activity of familial Alzheimer’s disease-associated mutant presenilin can be executed by six different γ-secretase complexes. Neurobiol. Dis..

[25] Bentahir M., Nyabi O., Verhamme J., Tolia A., Horré K., Wiltfang J. (2006). Presenilin clinical mutations can affect γ-secretase activity by different mechanisms. J. Neurochem..

[26] Lai M.T., Chen E., Crouthamel M.C., DiMuzio-Mower J., Xu M., Huang Q. (2003). Presenilin-1 and presenilin-2 exhibit distinct yet overlapping γ-secretase activities. J. Biol. Chem..

[27] Lee J., Song L., Terracina G., Bara T., Josien H., Asberom T. (2011). Identification of presenilin 1-selective γ-secretase inhibitors with reconstituted γ-secretase complexes. Biochemistry.

[28] Lessard C.B., Rodriguez E., Ladd T.B., Minter L.M., Osborne B.A., Miele L. (2020). γ-Secretase modulators exhibit selectivity for modulation of APP cleavage but inverse γ-secretase modulators do not. Alzheimers Res. Ther..

[29] Acx H., Chavez-Gutierrez L., Serneels L., Lismont S., Benurwar M., Elad N. (2014). Signature amyloid β profiles are produced by different γ-secretase complexes. J. Biol. Chem..

[30] Bai X.C., Rajendra E., Yang G., Shi Y., Scheres S.H. (2015). Sampling the conformational space of the catalytic subunit of human γ-secretase. Elife.

[31] Chen S.Y., Zacharias M. (2020). How mutations perturb γ-secretase active site studied by free energy simulations. ACS Chem. Neurosci..

[32] Dehury B., Tang N., Kepp K.P. (2019). Molecular dynamics of C99-bound γ-secretase reveal two binding modes with distinct compactness, stability, and active-site retention: implications for Aβ production. Biochem. J..

[33] Fukumori A., Feilen L.P., Steiner H. (2020). Substrate recruitment by γ-secretase. Semin. Cell Dev. Biol..

[34] Iwatsubo T., Odaka A., Suzuki N., Mizusawa H., Nukina N., Ihara Y. (1994). Visualization of Aβ42 (43) and Aβ40 in senile plaques with end-specific Aβ monoclonals: evidence that an initially deposited species is Aβ42 (43). Neuron.

[35] Cullen N., Janelidze S., Palmqvist S., Stomrud E., Mattsson-Carlgren N., Hansson O. (2022). Association of CSF Aβ38 levels with risk of Alzheimer disease-related decline. Neurology.

[36] Liu L., Lauro B.M., He A., Lee H., Bhattarai S., Wolfe M.S. (2023). Identification of the Aβ37/42 peptide ratio in CSF as an improved Aβ biomarker for Alzheimer's disease. Alzheimers Dement..

[37] Tagami S., Yanagida K., Kodama T.S., Takami M., Mizuta N., Oyama H. (2017). Semagacestat is a pseudo-inhibitor of γ-secretase. Cell Rep..

[38] Podlisny M.B., Citron M., Amarante P., Sherrington R., Xia W., Zhang J. (1997). Presenilin proteins undergo heterogeneous endoproteolysis between Thr291 and Ala299 and occur as stable N- and C-terminal fragments in normal and Alzheimer brain tissue. Neurobiol. Dis..

[39] Jacobsen H., Reinhardt D., Brockhaus M., Bur D., Kocyba C., Kurt H. (1999). The influence of endoproteolytic processing of familial Alzheimer's disease presenilin 2 on Aβ42 amyloid peptide formation. J. Biol. Chem..

[40] Steiner H., Capell A., Pesold B., Citron M., Kloetzel P.M., Selkoe D.J. (1998). Expression of Alzheimer’s disease-associated presenilin-1 is controlled by proteolytic degradation and complex formation. J. Biol. Chem..

[41] Wiltfang J., Smirnov A., Schnierstein B., Kelemen G., Matthies U., Klafki H.W. (1997). Improved electrophoretic separation and immunoblotting of beta-amyloid (Aβ) peptides 1-40, 1-42, and 1-43. Electrophoresis.

[42] Li Y.M., Lai M.T., Xu M., Huang Q., DiMuzio-Mower J., Sardana M.K. (2000). Presenilin 1 is linked with γ-secretase activity in the detergent solubilized state. Proc. Natl. Acad. Sci. U. S. A..

[43] May P., Altstiel L., Bender M., Boggs L., Calligaro D., Fuson K. (2001). Marked reduction of Aβ accumulation and β-amyloid plaque pathology in mice upon chronic treatment with a functional γ-secretase inhibitor. Soc. Neurosci. Abstr..

[44] Lessard C.B., Rodriguez E., Ladd T.B., Minter L.M., Osborne B.A., Miele L. (2019). Individual and combined presenilin 1 and 2 knockouts reveal that both have highly overlapping functions in HEK293T cells. J. Biol. Chem..

[45] Pinnix I., Musunuru U., Tun H., Sridharan A., Golde T., Eckman C. (2001). A novel γ-secretase assay based on detection of the putative C-terminal fragment-γ of amyloid β protein precursor. J. Biol. Chem..

[46] Kong R., Chang S., Xia W., Wong S.T. (2015). Molecular dynamics simulation study reveals potential substrate entry path into γ-secretase/presenilin-1. J. Struct. Biol..

[47] Tolia A., Horre K., De Strooper B. (2008). Transmembrane domain 9 of presenilin determines the dynamic conformation of the catalytic site of γ-secretase. J. Biol. Chem..

[48] Li X., Dang S., Yan C., Gong X., Wang J., Shi Y. (2013). Structure of a presenilin family intramembrane aspartate protease. Nature.

[49] Page R.M., Baumann K., Tomioka M., Perez-Revuelta B.I., Fukumori A., Jacobsen H. (2008). Generation of Aβ38 and Aβ42 is independently and differentially affected by familial Alzheimer disease-associated presenilin mutations and γ-secretase modulation. J. Biol. Chem..

[50] Kretner B., Fukumori A., Gutsmiedl A., Page R.M., Luebbers T., Galley G. (2011). Attenuated Aβ42 responses to low potency γ-secretase modulators can be overcome for many pathogenic presenilin mutants by second-generation compounds. J. Biol. Chem..

[51] Asami-Odaka A., Ishibashi Y., Kikuchi T., Kitada C., Suzuki N. (1995). Long amyloid beta-protein secreted from wild-type human neuroblastoma IMR-32 cells. Biochemistry.

[52] Saura C.A., Tomita T., Davenport F., Harris C.L., Iwatsubo T., Thinakaran G. (1999). Evidence that intramolecular associations between presenilin domains are obligatory for endoproteolytic processing. J. Biol. Chem..

[53] Watanabe H., Imaizumi K., Cai T., Zhou Z., Tomita T., Okano H. (2021). Flexible and accurate substrate processing with distinct presenilin/γ-secretases in human cortical neurons. eNeuro.

[54] Kakuda N., Funamoto S., Yagishita S., Takami M., Osawa S., Dohmae N. (2006). Equimolar production of amyloid β-protein and amyloid precursor protein intracellular domain from β-carboxyl-terminal fragment by γ-secretase. J. Biol. Chem..

[55] Mori K., Okochi M., Tagami S., Nakayama T., Yanagida K., Kodama T.S. (2010). The production ratios of AICDε51 and Aβ42 by intramembrane proteolysis of βAPP do not always change in parallel. Psychogeriatrics.

[56] Fukumori A., Okochi M., Tagami S., Jiang J., Itoh N., Nakayama T. (2006). Presenilin-dependent γ-secretase on plasma membrane and endosomes is functionally distinct. Biochemistry.

[57] Morishima-Kawashima M. (2014). Molecular mechanism of the intramembrane cleavage of the β-carboxyl terminal fragment of amyloid precursor protein by γ-secretase. Front. Physiol..

[58] Stromberg K., Hansson E.M., Laudon H., Bergstedt S., Naslund J., Lundkvist J. (2005). γ-Secretase complexes containing N- and C-terminal fragments of different presenilin origin retain normal γ-secretase activity. J. Neurochem..

[59] Watanabe N., Image I., II, Takagi S., Tominaga A., Image Image I., Tomita T. (2010). Functional analysis of the transmembrane domains of presenilin 1: participation of transmembrane domains 2 and 6 in the formation of initial substrate-binding site of γ-secretase. J. Biol. Chem..

[60] Corin K., Bowie J.U. (2020). How bilayer properties influence membrane protein folding. Protein Sci..

[61] Dehury B., Tang N., Blundell T.L., Kepp K.P. (2019). Structure and dynamics of γ-secretase with presenilin 2 compared to presenilin 1. RSC Adv..

[62] Cai T., Tomita T. (2020). Structure-activity relationship of presenilin in γ-secretase-mediated intramembrane cleavage. Semin. Cell Dev. Biol..

[63] Zhou R., Yang G., Guo X., Zhou Q., Lei J., Shi Y. (2019). Recognition of the amyloid precursor protein by human γ-secretase. Science.

[64] Cai T., Morishima K., Takagi-Niidome S., Tominaga A., Tomita T. (2019). Conformational dynamics of transmembrane domain 3 of presenilin 1 is associated with the trimming activity of γ-secretase. J. Neurosci..

[65] Cai T., Tomita T. (2021). Sequential conformational changes in transmembrane domains of presenilin 1 in Aβ42 downregulation. J. Biochem..

[66] Moehlmann T., Winkler E., Xia X., Edbauer D., Murrell J., Capell A. (2002). Presenilin-1 mutations of leucine 166 equally affect the generation of the Notch and APP intracellular domains independent of their effect on Aβ42 production. Proc. Natl. Acad. Sci. U. S. A..

[67] Fukumori A., Steiner H. (2016). Substrate recruitment of γ-secretase and mechanism of clinical presenilin mutations revealed by photoaffinity mapping. EMBO J..

[68] Trambauer J., Rodríguez Sarmiento R.M., Fukumori A., Feederle R., Baumann K., Steiner H. (2020). Aβ43-producing PS1 FAD mutants cause altered substrate interactions and respond to γ-secretase modulation. EMBO Rep..

[69] Tominaga A., Cai T., Takagi-Niidome S., Iwatsubo T., Tomita T. (2016). Conformational changes in transmembrane domain 4 of presenilin 1 are associated with altered Aβ42 production. J. Neurosci..

[70] Tomita T., Iwatsubo T. (2013). Structural biology of presenilins and signal peptide peptidases. J. Biol. Chem..

[71] Hitzenberger M., Zacharias M. (2019). Structural modeling of γ-secretase Aβn complex formation and substrate processing. ACS Chem. Neurosci..

[72] Aguayo-Ortiz R., Dominguez L. (2018). Simulating the γ-secretase enzyme: recent advances and future directions. Biochimie.

[73] Fersht A. (2006).

[74] Yang G., Zhou R., Zhou Q., Guo X., Yan C., Ke M. (2019). Structural basis of notch recognition by human γ-secretase. Nature.

[75] Guo X., Wang Y., Zhou J., Jin C., Wang J., Jia B. (2022). Molecular basis for isoform-selective inhibition of presenilin-1 by MRK-560. Nat. Commun..

[76] Kretner B., Trambauer J., Fukumori A., Mielke J., Kuhn P.-H., Kremmer E. (2016). Generation and deposition of A43 by the virtually inactive presenilin-1 L435F mutant contradicts the presenilin loss-of-function hypothesis of Alzheimer's disease. EMBO Mol. Med..

[77] Capell A., Saffrich R., Olivo J.C., Meyn L., Walter J., Grunberg J. (1997). Cellular expression and proteolytic processing of presenilin proteins is developmentally regulated during neuronal differentiation. J. Neurochem..

[78] Steiner H., Duff K., Capell A., Romig H., Grim M.G., Lincoln S. (1999). A loss of function mutation of presenilin-2 interferes with amyloid β-peptide production and Notch signaling. J. Biol. Chem..

[79] Yamasaki A., Eimer S., Okochi M., Smialowska A., Kaether C., Baumeister R. (2006). The GxGD motif of presenilin contributes to catalytic function and substrate identification of γ-secretase. J. Neurosci..

[80] Gibson D.G., Young L., Chuang R.-Y., Venter J.C., Hutchison C.A., Smith H.O. (2009). Enzymatic assembly of DNA molecules up to several hundred kilobases. Nat. Methods.

[81] Steiner H., Kostka M., Romig H., Basset G., Pesold B., Hardy J. (2000). Glycine 384 is required for presenilin-1 function and is conserved in bacterial polytopic aspartyl proteases. Nat. Cell Biol..

[82] Kretner B., Fukumori A., Kuhn P.H., Perez-Revuelta B.I., Lichtenthaler S.F., Haass C. (2013). Important functional role of residue x of the presenilin GxGD protease active site motif for APP substrate cleavage specificity and substrate selectivity of γ-secretase. J. Neurochem..

[83] Schagger H., von Jagow G. (1987). Tricine-sodium dodecyl sulfate-polyacrylamide gel electrophoresis for the separation of proteins in the range from 1 to 100 kDa. Anal. Biochem..

