# Supplementary material for: Different transmembrane domains determine the specificity and efficiency of the cleavage activity of the γ-secretase subunit presenilin
Source: J Biol Chem. 2023 Mar 20;299(5):104626. doi: 10.1016/j.jbc.2023.104626 (PMC10164903; doi:10.1016/j.jbc.2023.104626)
Supplement: Supporting Figures S1–S4 and Tables S1 and S2 [file mmc1.pdf]

Supporting Information

Different transmembrane domains determine the specificity and efficiency of the cleavage activity of the γ-secretase subunit presenilin

Fabian C. Schmidt[1], Katja Fitz[1,2], Lukas P. Feilen[3,4], Masayasu Okochi[5], Harald Steiner[3,6], and Dieter Langosch[1*]

[1] Chair of Biopolymer Chemistry, Technical University of Munich, Freising, Germany

[3] German Center for Neurodegenerative Diseases (DZNE), Munich, Germany

[5] Neuropsychiatry, Department of Integrated Medicine, Division of Internal Medicine, Osaka University Graduate School of Medicine, Suita, Japan

[6] Biomedical Center (BMC), Division of Metabolic Biochemistry, Faculty of Medicine, Ludwig-Maximilians-University, Munich, Germany

[2] current address: Institute of Biochemistry and Molecular Biology, University of Hamburg, Hamburg, Germany

[4] current address: Biomolecular Sciences, Department of Biology, University of Copenhagen, Copenhagen, Denmark

*Send correspondence to:

D. Langosch, Lehrstuhl für Chemie der Biopolymere, Technische Universität München, Weihenstephaner Berg 3, 85354 Freising, Germany. Tel.: +49-8161-71-3500; Fax: +49-8161-71-4404; E-mail: langosch@tum.de

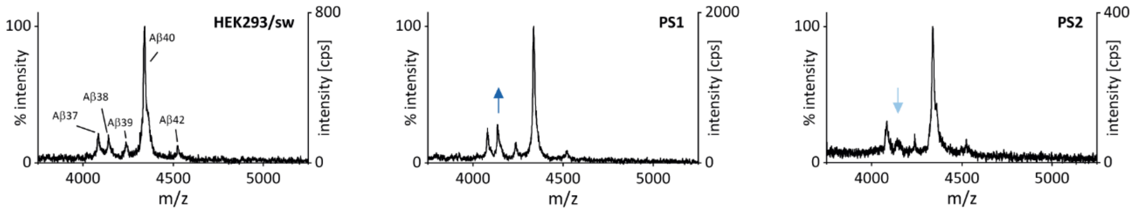

Figure S1. Comparing endogenous γ-secretase to PS1- and PS2-containing γ-secretases in terms of Aβ37 and Aβ38 production. The Aβ contents of conditioned media from HEK293/sw cells (left) or from pooled clones of HEK293/sw PS1/2$^{-/-}$ dKO cells stably transfected with PS1 (center) or PS2 (right) were analyzed by MALDI mass spectrometry following immunoprecipitation with antibody 4G8. The intensities of the highest peaks were set to 100%. Additionally, the counts per second (cps) are shown on the right y-axis. Arrows mark differences in Aβ38 production between PS1 and PS2.

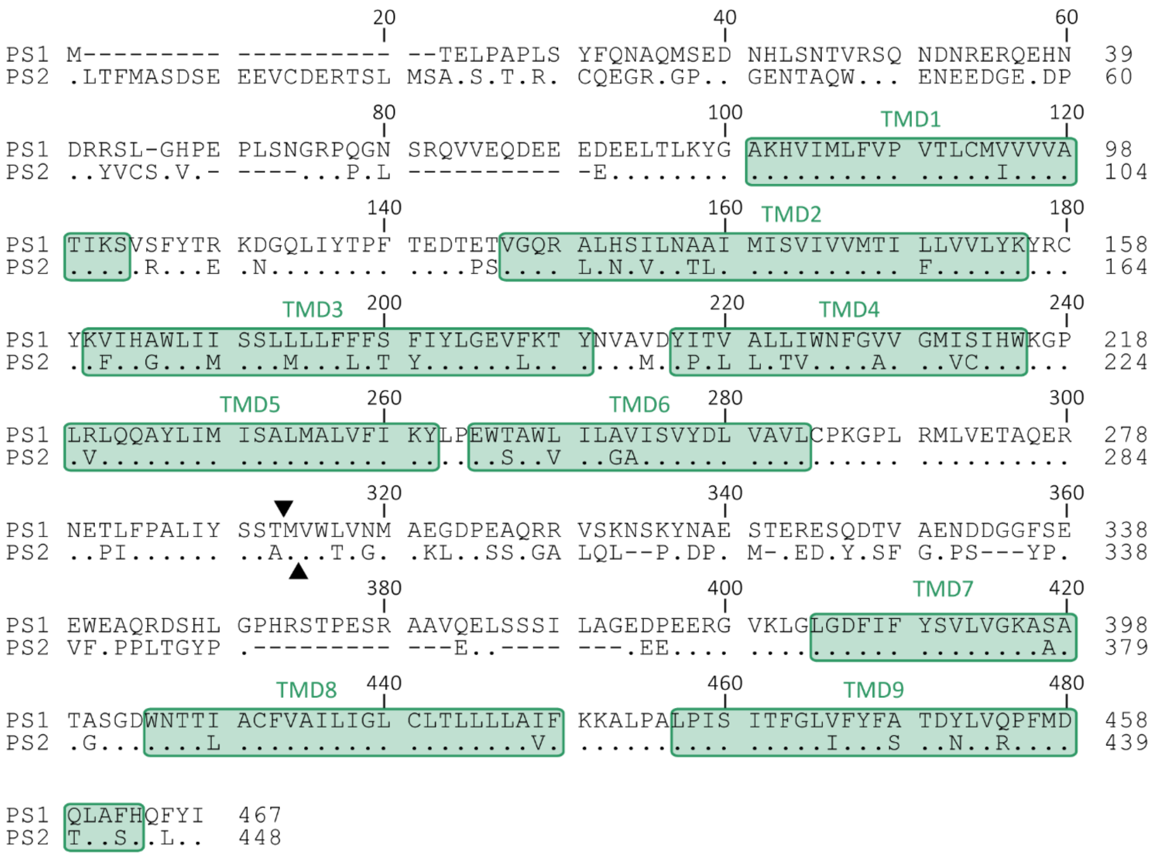

Figure S2. Sequence alignment of PS1 and PS2. Green boxes mark the TMDs according to the cryo-EM structure of γ-secretase at atomic resolution (PDB 5FN3 (1)). The endoproteolytic cleavage sites between TMD6 and TMD7 are indicated by black arrowheads. Dots and dashes represent conserved residues or gap positions, respectively. Sequences originate from UniProt and were aligned using CLC Main Workbench 6.

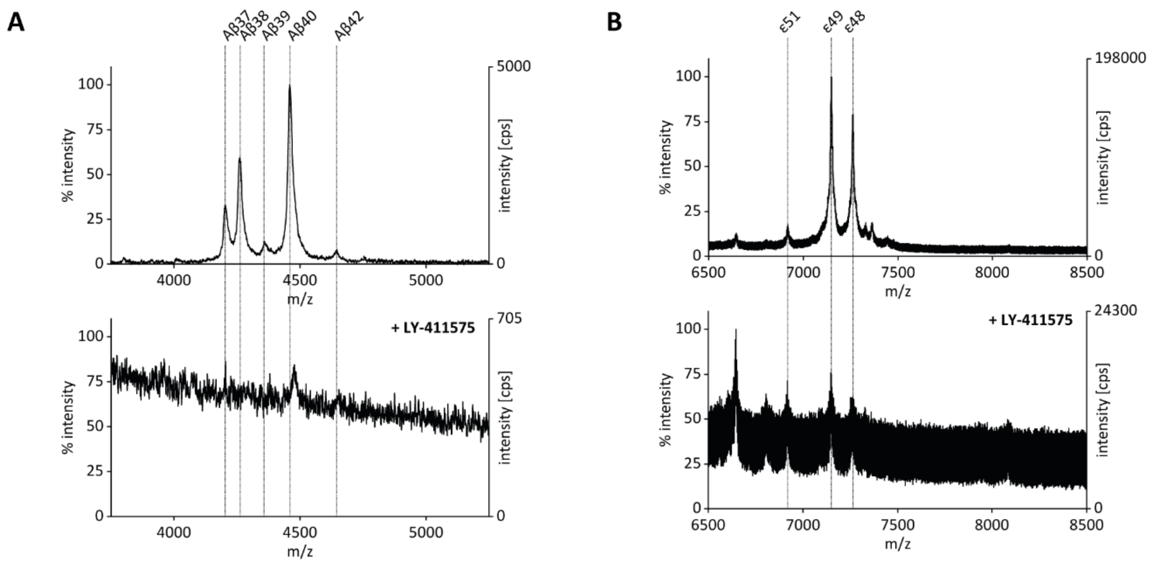

Figure S3. Verification of cleavage products detected by MALDI mass spectrometry as γ-secretase-specific. Cell-free assays using CHAPSO-solubilized membrane fractions with PS1-containing γ-secretase complexes and recombinant C100-His6 as a substrate were analyzed by MALDI mass spectrometry subsequent to immunoprecipitation with antibodies 4G8 for Aβ peptides **(A)** or Y188 (1:2500) for AICDs **(B)**. Control reactions with 1.22 µM LY-411575 γ-secretase inhibitor were analyzed in parallel (lower panels). Note the virtual absence of the corresponding signals of Aβ and AICD peptides in the lower panels, given their ca. 10-fold magnification (as indicated by the cps). The spectra in (B) were recorded with a different instrument than those in (A), resulting in higher overall cps.

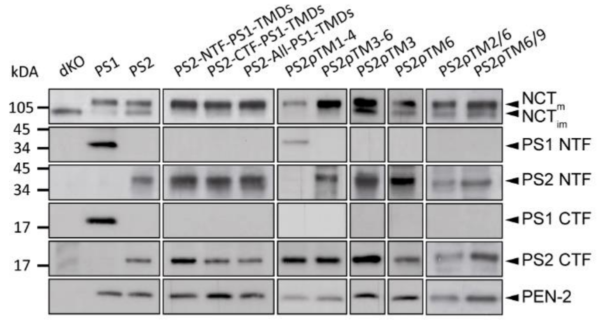

Figure S4. Maturation of γ-secretase containing the individual presenilin variants analyzed by immunoblotting its subunits. The analysis of detergent-solubilized membranes reveals the expected mass increase of NCT. This mass increase results from its N-glycosylation which converts non-assembled NCT_{im} to NCT_m being part of γ-secretase. Further, it documents NTF and CTF formation as a consequence of endoproteolytic presenilin cleavage. Both post-translational modifications are absent in dKO cells devoid of PS1 and PS2. Although expression levels of γ-secretase components are not uniform, they do not correlate with cleavage efficiency. For example, PS2ρTM1-4 has low levels of NCT and exhibits high activity, while the opposite is true for PS2ρTM3. Unfortunately, we found in the course of our study that some of the chimeric constructs used to study the specificity of cleavage, as presented in Fig. 2, exhibited non-trivial deviations in the levels of any γ-secretase subunit relative to wt presenilin (data not shown). Although varying concentrations of γ-secretase are unlikely to affect cleavage specificity, they might influence its efficiency. We therefore refrained from quantifying cleavage efficiencies of these aberrant chimeric constructs.

Table S1. List of investigated presenilin constructs.

Presenilin variant	Identity of fragments[1]
PS1	1-467 (PS1)
PS2	1-448 (PS2)
PS2ρTM3	1-161 (PS2), 156-194 (PS1), 201-448 (PS2)
PS2ρTM4	1-195 (PS2), 190-218 (PS1), 225-448 (PS2)
PS2ρTM6	1-246 (PS2), 241-291 (PS1), 299-448 (PS2)
PS2ρTM1-2	1-159 (PS1), 166-448 (PS2)
PS2ρTM3-4	1-161 (PS2), 156-218 (PS1), 225-448 (PS2)
PS2ρTM4-5	1-195 (PS2), 190-242(PS1), 249-448 (PS2)
PS2ρTM1-4	1-218 (PS1), 225-448 (PS2)
PS2ρTM3-6	1-161 (PS2), 156-291 (PS1), 299-448 (PS2)
PS2ρTM2/6	1-108 (PS2), 103-159 (PS1), 166-246 (PS2), 241-291 (PS1), 299-448 (PS2)
PS2ρTM6/9	1-246 (PS2), 241-291 (PS1), 299-409 (PS2); 429-467 (PS1)
PS1/2	1-291 (PS1), 299-448 (PS2)
PS2/1	1-298 (PS2), 292-467 (PS1)
PS2-All-PS1TMDs	1-84 (PS2), 79-105 (PS1), 109-130 (PS2), 125-155 (PS1), 162-165 (PS2), 160-189 (PS1), 196-200 (PS2), 195-215 (PS1), 222-224 (PS2), 219-240 (PS1), 247-248 (PS2), 243-252 (PS1), 259-363 (PS2), 383-398 (PS1), 380-384 (PS2), 404-428 (PS1), 410-415 (PS2), 435-463 (PS1), 445-448 (PS2)
PS2-NTF-PS1TMDs	1-84 (PS2), 79-105 (PS1), 109-130 (PS2), 125-155 (PS1), 162-165 (PS2), 160-189 (PS1), 196-200 (PS2), 195-215 (PS1), 222-224 (PS2), 219-240 (PS1), 247-248 (PS2), 243-252 (PS1), 259-448 (PS2)
PS2-CTF-PS1TMDs	1-363 (PS2), 383-398 (PS1), 380-384 (PS2), 404-428 (PS1), 410-415 (PS2), 435-463 (PS1), 445-448 (PS2)

[1] Chimeric presenilin variants were based on the PS2 template, which was fused with the given PS1 region(s). The identity of the individual fused fragments is indicated by the amino acid positions of PS1 or PS2, respectively. Annotations of membrane-spanning domains were obtained from the cryo-EM structure of γ-secretase at atomic resolution (PDB: 5fn3) (1).

Table S2. Mass and sequences of Aβ and AICD peptides generated in cell-free and cell-based assays as detected by MALDI mass spectrometry.

Peptide	Sequence	Calculated mass [Da]	Observed mass [Da]
Aβ37	DAEFRHDSGYEVHHQKLVFFAEDVGSNKGAIIGLMVG	4074.54	4073.89
Aβ38	DAEFRHDSGYEVHHQKLVFFAEDVGSNKGAIIGLMVGG	4131.59	4131.52
Aβ39	DAEFRHDSGYEVHHQKLVFFAEDVGSNKGAIIGLMVGGV	4230.72	4230.09
Aβ40	DAEFRHDSGYEVHHQKLVFFAEDVGSNKGAIIGLMVGGVV	4329.86	4331.20
Aβ42	DAEFRHDSGYEVHHQKLVFFAEDVGSNKGAIIGLMVGGVVIA	4514.10	4514.93
M-Aβ37[1]	MDAEFRHDSGYEVHHQKLVFFAEDVGSNKGAIIGLMVG	4205.73	4204.78
M-Aβ38[1]	MDAEFRHDSGYEVHHQKLVFFAEDVGSNKGAIIGLMVGG	4262.78	4263.33
M-Aβ39[1]	MDAEFRHDSGYEVHHQKLVFFAEDVGSNKGAIIGLMVGGV	4361.92	4360.65
M-Aβ40[1]	MDAEFRHDSGYEVHHQKLVFFAEDVGSNKGAIIGLMVGGVV	4461.05	4461.18
M-Aβ42[1]	MDAEFRHDSGYEVHHQKLVFFAEDVGSNKGAIIGLMVGGVVIA	4645.29	4647.62
AICDε51[2]	LKKKQYTSIHHGVVEVDAAVTPEERHLSKMQQNGYENPTYKFFEQMQNGS RSHHHHHH	6890.61	6918.33[3]
AICDε49[2]	VMLKKKQYTSIHHGVVEVDAAVTPEERHLSKMQQNGYENPTYKFFEQMQN GSRSHHHHHH	7120.94	7147.81[3]
AICDε48[2]	LVMLKKKQYTSIHHGVVEVDAAVTPEERHLSKMQQNGYENPTYKFFEQMQ NGSRSHHHHHH	7234.10	7262.39[3]

[1] All M-Aβ start with an additional N-terminal methionine derived from the C100-His_6 substrate used in cell-free assays.
[2] All AICD species contain a C-terminal hexa-histidine tag which is fused via a GSRS linker to the C100 substrate used in cell-free assays.
[3] observed masses correspond to AICD species with a sodium adduct $[AICD + Na]^+$.

Supplemental reference

30. X.C. Bai, E. Rajendra, G. Yang, Y. Shi and S.H. Scheres; Sampling the conformational space of the catalytic subunit of human γ-secretase, *Elife*, **4**, 2015, e11182.
